# Local Versus Systemic Tranexamic Acid in Total Hip Arthroplasty in Young Adults

**DOI:** 10.7759/cureus.36230

**Published:** 2023-03-16

**Authors:** Narendra S Kushwaha, Shubham Singh, Sanjiv Kumar, Arpit Singh, Mohammad Baqar Abbas, Siddharth Deshwal, Rishabh Agarwal

**Affiliations:** 1 Department of Orthopaedic Surgery, King George’s Medical University, Lucknow, IND

**Keywords:** total hip arthroplasty (tha), packed red blood cell transfusion, hip and knee replacement, postoperative blood loss, intravenous tranexamic acid

## Abstract

Background

Total hip arthroplasty (THA) is the most successful orthopedic elective surgical procedure for end-stage hip arthritis. THA is linked with significant blood loss, ranging from 1,188 to 1,651 mL, and a transfusion rate of 16-37%, which frequently results in postoperative blood transfusions. Postoperative blood transfusions can be avoided by using autologous blood transfusion, intraoperative blood saving, local anesthetic, hypotensive anesthesia, and antifibrinolytic medications such as tranexamic acid (TXA) administration.

Methodology

A double-blinded, placebo-controlled, randomized, controlled study was conducted with three prospective groups to investigate the efficacy of topical and systemic routes of a single intraoperative dose (1.5 g) of TXA. Patients were recruited from our center between October 2021 to March 2022 who were undergoing primary total hip replacement. Estimated blood loss was calculated and compared in groups, and a p-value of <0.05 was taken as significant.

Results

A total of 60 patients were recruited in our study. Estimated blood loss was similar in both treatment groups, 816.8 ± 219.9 mL in the systemic TXA group and 775.5 ± 107.2 mL in the topical TXA group. The placebo group had 1,066.3 ± 150.4 mL estimated blood loss, which was significantly higher compared to the treatment groups.

Conclusions

Administration of TXA (1.5 g) significantly lowers blood loss without increasing problems, which can eliminate concerns about intravenous TXA use. TXA reduces blood loss by 270 mL on average.

## Introduction

Total hip arthroplasty (THA) is one of the most successful orthopedic elective surgery for end-stage hip arthritis [1]. THA has several advantages and greatly improves patients’ quality of life. Patients can perform a wide range of daily activities due to painless hip motions, allowing them to live a normal life. THA is associated with considerable blood loss of between 1,188 ml and 1,651 mL [2] and a transfusion rate as high as 16-37%, as reported in the literature [3,4]. The risks and negative effects of perioperative transfusions include the transmission of infectious organisms, hemolytic transfusion reactions, and short-term mortality [5,6]. Postoperative blood transfusions can be avoided by using autologous blood transfusion, intraoperative blood saving, local anesthetic, hypotensive anesthesia, and antifibrinolytic medications such as tranexamic acid (TXA) administration [7]. TXA, a synthetic lysine derivative, lowers fibrinolysis activity by competitively obstructing lysine binding sites on plasminogen molecules. The body’s capacity to hold onto blood clots is enhanced by TXA. As a result, bleeding is reduced more effectively. The effectiveness of intravenous TXA has been studied in several THA trials [8,9] and meta-analyses, which have shown it to be effective. TXA may reduce transfusions and blood loss after THA without raising thromboembolic events [10,11]. Recently, total knee arthroplasty has seen a rise in the local use of TXA, which has been shown to reduce blood loss. On the other hand, the effectiveness of TXA in THA is still up for debate. While some studies found that TXA reduced blood loss more effectively [12-21], others found no differences in the rates of transfusion between the two groups [22-25]. This study aimed to determine whether local TXA reduced blood loss during hip replacement surgery better than systemic TXA.

## Materials and methods

Three prospective groups participated in the study to examine the effectiveness of topical and systemic delivery of a single intraoperative dose (1.5 g) of TXA. For the systemic TXA, local TXA, and placebo groups, the allocation ratios were 1:1:1. Patients receiving primary total hip replacements were included from our center between October 2021 and March 2022. Patients were not included if they had a history of thrombosis or a risk of developing it, refused blood transfusions, had a known allergy to TXA, had an active thromboembolic disease, were pregnant or nursing, were on anticoagulant therapy within five days of surgery, or had severe renal failure. Surgery was performed by a single surgeon using the modified Hardinge approach. The study drug was administered intracapsular after the capsule was closed, followed by the usual closure in layers. In addition to injecting the intracapsular study drug, the anesthesiologist also administered the intravenous study drug, as mentioned in Table 1.

**Table 1:** Study groups. TXA = tranexamic acid

Group	Intervention
A	Intracapsular: After placement of the implant and closure of the capsulotomy, 20 mL of normal saline is injected intracapsular, followed by standard closure. Intravenous: 1.5 g TXA
B	Intracapsular: After placement of the implant and closure of the capsulotomy, 1.5 g TXA in 20 mL is injected intracapsular, followed by standard closure. Intravenous: 20 mL normal saline
C	Intracapsular: After placement of the implant and closure of the capsulotomy, 20 mL of normal saline is injected intracapsular, followed by standard closure. Intravenous: 20 mL normal saline

Estimated blood loss was calculated using the difference between preoperative hemoglobin (Hb) and the final Hb before discharge, or, at the latest, day three (EBL). The formula by Good et al. was used to calculate the EBL [26]. The amount of Hb lost (in g) was determined using the following formula: Hbloss = BV × (Hbi - Hbe) × 0.001 + Hbt, where Hbi (g/L) is the Hb levels before the surgery, Hbe (g/L) is the Hb levels on the third day following the surgery, and Hbt (g) represents the total amount of allogeneic Hb transfused. Hbloss (g) represents the amount of Hb lost. Using Nadler’s method, which takes into account the patient’s gender, weight, and height [27], the estimated blood volume (mL) was computed. A blood bank unit was thought to have at least 35 g of Hb: Estimated blood loss = 1,000 × Hbloss / Hbi.

A Hb of 80 g/L was considered to be an indication that blood products should be transfused.

Measurements included transfusion rates, the average length of stay, 30-day readmissions, and complications. Deep vein thrombosis (DVT) with symptoms, pulmonary embolism (PE), and infection were considered to be complications.

The sequences were obtained by a separate biostatistician. After that, pre-programmed group instructions for the placebo, intracapsular, and systemic groups were each given the sequence in a 1:1:1 ratio. These were then randomly split into three groups using computerized randomization.

The chi-square test, one-way analysis of variance (ANOVA), followed by Tukey post hoc test, and paired t-test were used to analyze the variables.

## Results

A total of 60 patients were recruited in our study. The demographic and clinical characteristics are presented in Table 2. The most common indication for primary THA was avascular necrosis (AVN) of the head of the femur. In total, 36 patients in our study had AVN, eight had infective arthritis of the hip joint, seven had systemic arthritis (AS/RA) with secondary osteoarthritis (OA) of the hip joint, three had post-traumatic arthritis, and six had failed fracture neck femur as the diagnosis. The most common cause of AVN of the head of the femur was idiopathic in 18 patients, followed by steroid intake in seven, smoking/alcoholism in eight, and post-traumatic (hip dislocation/fracture neck femur) in three.

**Table 2:** Demographics

Patient characteristics	A (n = 20)	B (n = 20)	C (n = 20)
Age (years)	39.20 ± 10.78	44.00 ± 16.12	41.30 ± 15.13
Gender (male:female)	14:6	10:10	12:8

Preoperative mean Hb level was 133.4 ± 16.26 g/L in group A, 131.8 ± 14.83 g/L in group B, and 129.9 ± 14.85 g/L in group C. No significant difference was found in the mean Hb level among the groups (p = 0.771).

**Figure 1:**
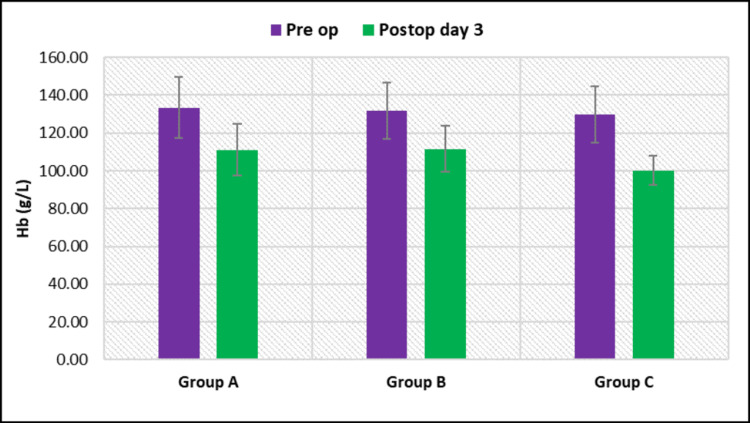
Intergroup and intragroup comparison of hemoglobin (Hb) levels.

At postoperative day three, the mean Hb level was 111.0 ± 13.70 g/L in group A, 111.5 ± 12.18 g/L in group B, and 100.20 ± 7.67 g/L in group C. A significant difference was found in the mean Hb level among the groups (p = 0.004). It was the maximum in group B and the minimum in group C (Figure 1).

Further intragroup comparisons showed significant changes in group A (p < 0.001), group B (p < 0.001), and group C (p < 0.001) (Table 3).

**Table 3:** Intergroup and intragroup comparison of hemoglobin levels. ANOVA = analysis of variance; Hb = hemoglobin

Group	Group A		Group B		Group C		ANOVA	
	Mean	SD	Mean	SD	Mean	SD	F-value	P-value
Preoperative Hb (g/L)	133.40	16.26	131.80	14.83	129.90	14.85	0.26	0.771
Postoperative day three Hb	111.00	13.70	111.50	12.18	100.20	7.67	6.19	0.004
Intragroup	t = 14.713, p < 0.001		t = 24.723, p < 0.001		t = 16.881, p < 0.001			

Comparison of Hb levels on day three between pairs of groups showed that there was a significant difference between group A and group C (p = 0.012) and between group B and group C (p = 0.008). However, no significant difference was found between group A and group B (p = 0.990) (Table 4).

**Table 4:** Pairwise comparison of hemoglobin levels on day three. Hb = hemoglobin; SE = standard error

Group pair		Postoperative day three Hb		
		Mean difference	SE	P-value
Group A	Group B	-0.50	3.63	0.990
Group A	Group C	10.80	3.63	0.012
Group B	Group C	11.30	3.63	0.008

Analysis of outcomes and complications of study groups is presented in Table 5.

**Table 5:** Secondary outcomes and complications. DVT = deep vein thrombosis; PE = pulmonary embolism

Outcomes and complications	A (n = 20)	B (n = 20)	C (n =20)	P-value
Perioperative fluids (mL)	1,575.00	1,250.00	1,525.00	0.002
Blood transfused (yes:no)	4:16	4:16	10:10	-
Number of units transfused	1	1	1.4	0.143
Length of stay	7.20	7.90	8.70	0.563
DVT	0	0	0	-
PE	0	0	0	-
Superficial wound infection	4	5	4	-

**Figure 2:**
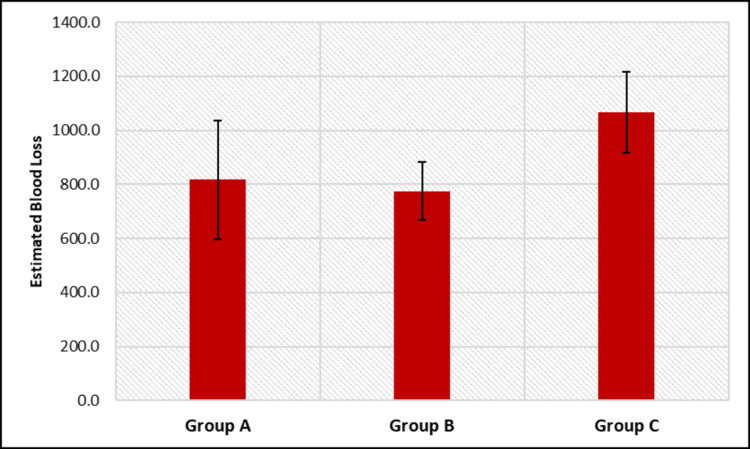
Intergroup comparison of estimated blood loss.

Estimated blood loss was the maximum in group C and was found to be statistically significant when compared with other groups (Table 6, Figure 2).

**Table 6:** Intergroup comparison of estimated blood loss. ANOVA = analysis of variance

Group	Estimated blood loss (mL)		One-way ANOVA	
	Mean	SD	F-value	P-value
Group A	816.8	219.9	18.01	<0.001
Group B	775.5	107.2		
Group C	1,066.3	150.4		

The post hoc comparison of estimated blood loss between pairs of groups showed that there was a significant difference between group A and group C (p < 0.001) and between group B and group C (p < 0.001). However, no significant difference was found between group A and group C (p = 0.712) (Table 7).

**Table 7:** Paired comparisons of estimated blood loss.

Group pair		Estimated blood loss	
		Mean difference	P-value
Group A	Group B	41.30	0.712
Group A	Group C	-249.53	<0.001
Group B	Group C	-290.84	<0.001

## Discussion

TXA in various studies has shown a reduction in postoperative blood loss and the need for blood transfusions in hip and knee arthroplasty [10,20,25]. Interest has been aroused by recent studies assessing the effectiveness of topical TXA administration in knee and hip arthroplasty [12,20]. In this study, we compared the topical administration of TXA with the intravenous route and placebo in light of the evidence for systemic TXA. TXA is a synthetic derivative of the amino acid lysine that inhibits the production of plasmin by blocking the lysine binding sites on plasminogen molecules [28]. A thorough review and meta-analysis have shown that intravenous TXA is effective in reducing transfusion rates and blood loss in THA [10]. The potential for thromboembolism in high-risk patients undergoing arthroplasty and the safety of systemic TXA administration remains a concern. After joint replacement, topical TXA therapy may be a safer delivery technique with comparable efficacy but much reduced systemic absorption and, thus, a lower risk of thromboembolic complications [29]. TXA was administered topically, which had the benefit of directly targeting the surgical site with the maximum concentration of TXA in the joint without systemic side effects. There is currently a dearth of information on the use of topical TXA in THA, despite recent meta-analyses supporting the use of intravenous TXA to reduce postoperative blood loss in THA [20].

Estimated blood loss was our primary outcome [26]. Patients receiving TXA experienced roughly 270 mL less blood loss on average regardless of the administration route than patients who did not receive TXA. There were significant differences in blood loss between the topical TXA and placebo groups in this analysis and between the systemic TXA and placebo groups. Apart from patients who are at high risk of cardiac and thromboembolic problems, topical administration of TXA is a good alternative delivery method and might even be superior for all patients undergoing hip replacement. In a double-blind, randomized controlled trial, Alshryda et al. examined the impact of topical TXA on blood loss in 161 patients undergoing THA and discovered that it was effective in reducing blood loss and the need for blood transfusions [20]. To assess the impact of topical TXA in THA, Yue et al. also conducted a randomized double-blind controlled experiment [19]. Their findings revealed that 3 g of topical TXA may significantly lower blood loss, drain output, transfusion rates, and Hb levels.

This study has several limitations. The success of topical TXA relies heavily on the watertight closure of the capsulotomy wound. As a result, the blood loss seen in this study may be underestimated. Estimated blood loss is calculated using an indirect method that involves preoperative Hb and serial postoperative Hb readings.

## Conclusions

This study found that topical administration of TXA (1.5 g) significantly lowers blood loss without increasing problems, which can eliminate concerns about intravenous TXA use. TXA lowers blood loss by 270 mL on average. We recommend the topical use of TXA in THA cases to decrease blood loss. Evidence also supports the use of TXA to reduce blood loss and the risk of blood transfusion after primary THA.

## References

[1] Iorio R, Robb WJ, Healy WL (2008). Orthopaedic surgeon workforce and volume assessment for total hip and knee replacement in the United States: preparing for an epidemic. J Bone Joint Surg Am.

[2] Rosencher N, Kerkkamp HE, Macheras G (2003). Orthopedic Surgery Transfusion Hemoglobin European Overview (OSTHEO) study: blood management in elective knee and hip arthroplasty in Europe. Transfusion.

[3] Bierbaum BE, Callaghan JJ, Galante JO, Rubash HE, Tooms RE, Welch RB (1999). An analysis of blood management in patients having a total hip or knee arthroplasty. J Bone Joint Surg Am.

[4] Gibon E, Courpied JP, Hamadouche M (2013). Total joint replacement and blood loss: what is the best equation?. Int Orthop.

[5] Vamvakas EC, Blajchman MA (2009). Transfusion-related mortality: the ongoing risks of allogeneic blood transfusion and the available strategies for their prevention. Blood.

[6] Carson JL, Grossman BJ, Kleinman S (2012). Red blood cell transfusion: a clinical practice guideline from the AABB*. Ann Intern Med.

[7] Watts CD, Pagnano MW (2012). Minimising blood loss and transfusion in contemporary hip and knee arthroplasty. J Bone Joint Surg Br.

[8] Poeran J, Rasul R, Suzuki S (2014). Tranexamic acid use and postoperative outcomes in patients undergoing total hip or knee arthroplasty in the United States: retrospective analysis of effectiveness and safety. BMJ.

[9] Clavé A, Fazilleau F, Dumser D, Lacroix J (2012). Efficacy of tranexamic acid on blood loss after primary cementless total hip replacement with rivaroxaban thromboprophylaxis: a case-control study in 70 patients. Orthop Traumatol Surg Res.

[10] Sukeik M, Alshryda S, Haddad FS, Mason JM (2011). Systematic review and meta-analysis of the use of tranexamic acid in total hip replacement. J Bone Joint Surg Br.

[11] Zhou XD, Tao LJ, Li J, Wu LD (2013). Do we really need tranexamic acid in total hip arthroplasty? A meta-analysis of nineteen randomized controlled trials. Arch Orthop Trauma Surg.

[12] Chang CH, Chang Y, Chen DW, Ueng SW, Lee MS (2014). Topical tranexamic acid reduces blood loss and transfusion rates associated with primary total hip arthroplasty. Clin Orthop Relat Res.

[13] Fan FC, Gui BJ (2014). Tranexamic acid injection through articular cavity and discontinuous clip pipe after total hip arthroplasty: changes in bleeding amount. Chin J Tissue Eng Res.

[14] Gilbody J, Dhotar HS, Perruccio AV, Davey JR (2014). Topical tranexamic acid reduces transfusion rates in total hip and knee arthroplasty. J Arthroplasty.

[15] Konig G, Hamlin BR, Waters JH (2013). Topical tranexamic acid reduces blood loss and transfusion rates in total hip and total knee arthroplasty. J Arthroplasty.

[16] Van Elst CE, Vanbiervliet J, Simon JP (2013). The Effect of Topical Application of Tranexamic Acid in Total Hip Arthroplasty Through the Direct Anterior Approach.

[17] Wei W, Wei B (2014). Comparison of topical and intravenous tranexamic acid on blood loss and transfusion rates in total hip arthroplasty. J Arthroplasty.

[18] Yin H, Wang DW, Ma WP, Xing DL, W ZF (2014). The efficacy and safety of tranexamic acid for reducing blood loss in primary total hip arthroplasty. Chin J Joint Surg.

[19] Yue C, Kang P, Yang P, Xie J, Pei F (2014). Topical application of tranexamic acid in primary total hip arthroplasty: a randomized double-blind controlled trial. J Arthroplasty.

[20] Alshryda S, Mason J, Sarda P (2013). Topical (intra-articular) tranexamic acid reduces blood loss and transfusion rates following total hip replacement: a randomized controlled trial (TRANX-H). J Bone Joint Surg Am.

[21] Ding M, QI W, Liu F, XU Z, GU Y, Lin M (2014). Efficacy of topical tranexamic acid in total hip arthroplasty. J Med Postgrad.

[22] Bagsby DT, Hur J (2014). Effect of intra-articular injection of tranexamic acid on postoperative hemoglobin in total hip arthroplasty. Orthopedics.

[23] Martin JG, Cassatt KB, Kincaid-Cinnamon KA, Westendorf DS, Garton AS, Lemke JH (2014). Topical administration of tranexamic acid in primary total hip and total knee arthroplasty. J Arthroplasty.

[24] Machin JT, Batta V, Soler JA, Sivagaganam K, Kalairajah Y (2014). Comparison of intra-operative regimes of tranexamic acid administration in primary total hip replacement. Acta Orthop Belg.

[25] Wind TC, Barfield WR, Moskal JT (2014). The effect of tranexamic acid on transfusion rate in primary total hip arthroplasty. J Arthroplasty.

[26] Good L, Peterson E, Lisander B (2003). Tranexamic acid decreases external blood loss but not hidden blood loss in total knee replacement. Br J Anaesth.

[27] Nadler SB, Hidalgo JH, Bloch T (1962). Prediction of blood volume in normal human adults. Surgery.

[28] Astedt B (1987). Clinical pharmacology of tranexamic acid. Scand J Gastroenterol Suppl.

[29] Wong J, Abrishami A, El Beheiry H (2010). Topical application of tranexamic acid reduces postoperative blood loss in total knee arthroplasty: a randomized, controlled trial. J Bone Joint Surg Am.

